# Role of *swi7H4* Mutant Allele of DNA Polymerase α in the DNA Damage Checkpoint Response

**DOI:** 10.1371/journal.pone.0124063

**Published:** 2015-03-30

**Authors:** Saman Khan, Shakil Ahmed

**Affiliations:** Molecular and Structural Biology Division, CSIR- Central Drug Research Institute, Jankipuram Extension, Lucknow, India; The University of Hong Kong, HONG KONG

## Abstract

Besides being a mediator of initiation of DNA replication, DNA polymerase α plays a key role in chromosome maintenance. *Swi7H4*, a novel temperature sensitive mutant of DNA polymerase α was shown to be defective in transcriptional silencing at the mating type centromere and telomere loci. It is also required for the establishment of chromatin state that can recruit the components of the heterochromatin machinery at these regions. Recently the role of DNA polymerase α in the S-phase alkylation damage response in *S*. *pombe* has also been studied. Here we investigate whether defects generated by s*wi7H4*, a mutant allele of DNA polymerase α can activate a checkpoint response. We show that s*wi7H4* exhibit conditional synthetic lethality with *chk1* null mutant and the double mutant of s*wi7H4* with *chk1* deletion aggravate the chromosome segregation defects. More importantly s*wi7H4* mutant cells delay the mitotic progression at non permissive temperature that is mediated by checkpoint protein kinase Chk1. In addition we show that, in the s*wi7H4* mutant background, cells accumulate DNA damage at non permissive temperature activating the checkpoint kinase protein Chk1. Further, we observed synthetic lethality between s*wi7H4* and a number of genes involved in DNA repair pathway at semi permissive temperature. We summarize that defects in s*wi7H4* mutant results in DNA damage that delay mitosis in a Chk1 dependent manner that also require the damage repair pathway for proper recovery.

## Introduction

Faithful chromosomal segregation is precisely monitored by the array of proteins involved in cell cycle events, like DNA replication, repair, mitosis and cytokinesis. Checkpoints monitor key events during the cell cycle and block subsequent event if earlier ones are incomplete and thus ensure the accuracy of each step in the cell cycle [1]. The cell maximizes its efforts of survival by coordinating with these proteins. DNA replication is the process through which a cell passes its genetic information from one generation to another. Hence the DNA replication machinery must be properly governed and protected from any genotoxic agent. In response to genotoxic assault the cell cycle abort, DNA repair machinery takes over and if the defects are still not repaired, then the cells undergo apoptosis. DNA damage response (DDR) senses different types of DNA damage and transmit the signal to activate the cell cycle checkpoint response [2]. Checkpoint pathways consist of various sensor, transducer and effector molecules. Sensors protein includes MRN complex (*mre11*, *rad 50* and *nbs1*) that recognises DNA double strand break [3] while Rad17 and Rad9-Hus1-rad1 (9-1-1) complex recognises replication stress [4]. Sensor molecules further activate transducer kinases, ATM (Ataxia-telangiectasia mutated) and ATR (ATM and Rad3-related). The Tel1/Rad3 in *S*. *pombe* and Tel1/Mec1 in *S*. *cerevisiae* are responsible for the activation of the effector kinases, Chk1 and Chk2 [5].

DNA polymerases are a family of enzymes that are responsible for all forms of DNA replication [6]. DNA polymerases ensure high fidelity of DNA replication through a balance between polymerase and the proof reading activities. In vitro reconstitution studies have demonstrated that three proteins are required for the initiation of DNA synthesis on duplex DNA molecules. These include the DNA polymerase α-primase complex, replication protein A, and T antigen [7,8]. DNA polymerase α was the first polymerase identified in eukaryotic cells and for several years it was thought to be the only polymerase required for chromosomal DNA replication [9]. Mutation in the polα gene affects several cellular processes such as DNA repair and recombination [10,11], transcriptional silencing [12,13], checkpoint activation [14,15] and telomere length maintenance [16–19]. Recently it is also reported that DNA polymerase α (*swi7*) and the flap endonuclease Fen1 (rad2) act together in the S-phase alkylation damage response in *S*. *pombe* [20].

A temperature sensitive mutant allele of DNA polymerase α, *swi7H4* is defective in silencing at centromere, telomere and mating type loci [12,13]. Further, DNA polymerase α was shown to be required for replication mediated recruitment of Swi6 to heterochromatin [13]. Fission yeast Cds1, a critical transducer of the replication checkpoint was isolated as multi-copy suppressor of *swi7H4* [21] indicating a connection between replication machinery and checkpoint response.

In this study, we elucidated the effect of *swi7H4* mutant on cell cycle progression. A conditional synthetic lethality of *swi7H4* mutant with *chk1* deletion was observed and the double mutant of *swi7H4* with *chk1* deletion aggravates the chromosome segregation defects. Further *swi7H*4 mutant cells delay the mitotic progression at non permissive temperature in a Chk1 dependent manner. More importantly, *swi7H4* mutant cells accumulate DNA damage at non permissive temperature and hence activate the checkpoint kinase protein Chk1. The genetic interaction of *swi7H4* with a number of genes involved in DNA repair pathway was also studied.

## Materials and Methods

### Strains and growth condition

*Schizosaccharomyces pombe* strains used in this study are listed in Table 1. Standard genetic methods were utilized for making strains as described in [22]. Standard yeast protocols were used for strain construction, growth and medium preparation as described in [23]. For temperature-shift experiments, cells were grown to mid log phase at 25°C, 10^7^ cells were serially diluted and spotted on YEA plates and incubated at the indicated temperature. For survival assay, the mid log phase culture was shifted at 36°C, samples were collected at different time intervals, 1000 cells from each sample were plated on YEA plates and incubated at 25°C until colonies appear. Colonies were counted and graph was plotted. For passing mitosis experiments, cells were synchronized in S phase by growing them in 12 mM hydroxyurea (HU) for 4 h at 25°C, washed and released at 36°C. The samples were collected at a 15 min interval and the number of cells having two nuclei was counted as an indication of cells entering into mitosis as described previously [24].

**Table 1:** Strains used in this study.

Strain	Genotype	Source
SP6	*h* ^*-*^ *leu1-32*	Lab stock
NW158	*h* ^*+*^ *leu1-32 ura4D18 chk1*::*ura4 ade6-216*	Nancy Walworth
NW223	*h* ^*+*^ *leu1-32 chk1*.*ep ade6-216*	Nancy Walworth
NW1497	*h* ^*-*^ *leu1-32 ura4D18 rad22-YFP-kan* ^*R*^	Nancy Walworth
TMP712	*h* ^*+*^ *leu-32 ura4D18 rhp51*::*ura4 ade6* ^*-*^	Nancy Walworth
1590	*h* ^*+*^ *leu1-32 ura4D18 hus2*::*ura4*	Nancy Walworth
NB2554	*h* ^*-*^ *ura4D18 Mus81*::*kan* ^*R*^	Nancy Walworth
1196	*h* ^*+*^ *leu1-32 ura4D18 rad2*::*ura4 ade6-704*	Nancy Walworth
SH318	*h* ^*+*^ *leu1-32 ura4D18 rad50*::*ura4 ade6-216*	Jagmohan Singh
SH217	*h- leu1-32 swi7H4 ade6-210*	This study
SH167	*h+ leu1-32 ura4D18 swi7H4 chk1*::*ura4 ade6-216*	This study
SH175	*h- leu1-32 ura4D18 swi7H4 chk1*.*ep ade6-216*	This study
SH551	*h leu1-32 ura4D18 swi7H4 rad22-YFP-KanR ade6* ^*-*^	This study
SH635	*h leu1-32 ura4D18 swi7H4 rhp51*::*ura4 ade6* ^*-*^	This study
SH643	*h leu1-32 ura4D18 swi7H4 hus2*::*ura4 ade6-216*	This study
SH639	*h leu1-32 swi7H4 mus81*::*kan* ^*R*^ *ade6-210*	This study
SH636	*h leu1-32 ura4D18 swi7H4 rad50*::*ura4 ade6-216*	This study
SH637	*h leu1-32 ura4D18 swi7H4 rad2*::*ura4 ade6-216*	This study

### Fluorescence Microscopy

For staining nuclei, cells were grown to mid-log phase at 25°C then shifted to 36°C, samples were collected at the indicated times, fixed with 70% ethanol and stained with DAPI(4’,6- diamidino-2-phenylindole), visualized using a fluorescence microscope. About 200 cells were counted for aberrant nuclei and their percentage was plotted.

### Preparation of lysates and Western-blot analysis

Cells containing Chk1 tagged with triple HA were grown to mid-log phase at 25°C then shifted at 36°C in water bath. Cells were harvested at different time intervals by centrifugation and lysed using glass beads and fast prep cell disruptor (MP Biosciences). Lysate was prepared as described in [23]. For Western-blot analysis, about 200 mg of total cell lysate was run on 8% SDS-PAGE, transferred to nitrocellulose membrane and probed with anti HA antibody (F7).

### Immunofluorescence

Immunofluorescence studies were performed using exponentially growing cells, essentially as described earlier [25]. The cells were harvested, washed and re-suspended in PEMS containing zymolyase. The samples were washed gently with 1X PEMS containing 1% triton and the cell pellet was re-suspended in 1 ml PEMBAL and further incubated at room temperature for 1hr. Rad22-YFP was detected using anti GFP antibody at 1:50 dilution and Alexa fluor 488 (Life Technologies) secondary antibody. Approximately 200 cells were analyzed using fluorescence microscope and the number of cells containing Rad22 YFP foci was counted.

### Flow Cytometry

Strains were grown till mid log phase, synchronized in early S phase by treating with 12mM hydroxyurea (HU) for 4 hr at 25°C, washed and released at 36°C. Aliquots of 10^6^ cells were collected, fixed with 70% ethanol before. For flow cytometry, rehydrated samples were re-suspended in 0.5 ml of 50mM sodium citrate containing 0.1 mg/ml RNase A and incubated at 37°C for 2h. Cells were stained by adding 0.5 ml of sodium citrate solution containing 10μg/ml Propidium Iodide and subjected to flow cytometry as described earlier [26]. Just prior to flow cytometry, samples were sonicated to avoid inaccurate readings resulting from the clumping of cells. Samples were analyzed with a Becton-Dickinson FACS Calibur.

## Results and Discussion

### Swi7H4 exhibit conditional synthetic lethality with Chk1 null mutant

**Fig 1:**
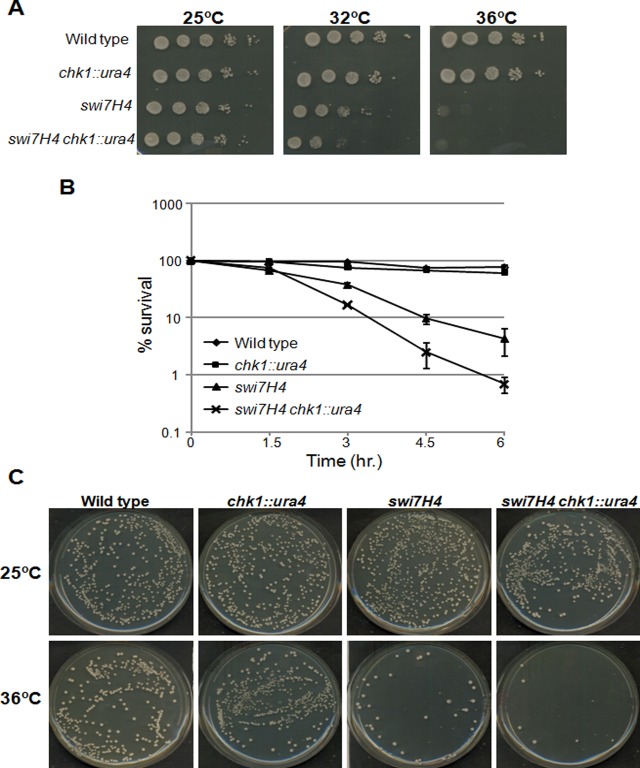
DNA polα mutant, *swi7H4* exhibit conditional lethality with *chk1* knockout. (A) Indicated strains were grown at 25°C, serially diluted and spotted on YEA plates. Plates were incubated at indicated temperature for 3 days before taking photographs. (B) Percent Survival of *swi7H4 chk1* knockout strain at non permissive temperature: Indicated strains were grown till mid log phase at 25°C then shifted at 36°C. Sample were collected at 1.5 hour intervals, equal number of cells were plated on YEA plates and incubated at 25°C. Number of surviving colonies was calculated as the percentage of colonies, appearing at permissive temperature. Values shown are the average of three independent experiments with standard deviation. (C) The plates showing surviving colonies from figure B at time point 0 (upper panel) and 6hr at 36°C (lower panel) were photographed.

*swi7H4*, a temperature sensitive mutant allele of DNA polymerase α is required for the establishment of silencing in fission yeast [12,13]. Since this mutant allele exhibit elongated phenotype (an indicator of G2/M arrest) at non permissive temperature, we tried to explore its genetic interaction with Chk1, a G2/M checkpoint kinase protein. A double mutant of *swi7H4 chk1* knockout was constructed and its ability to grow at different temperatures was assayed. The *swi7H4* mutant cells were unable to form colonies at non permissive temperature 36°C. Interestingly the colony forming ability of *swi7H4 chk1* delete cells was much lower than *swi7H4* single mutant at semi-permissive temperature 32°C (Fig 1A) indicating conditional synthetic lethality of *swi7H4* mutant with *chk1* null mutant. To further confirm the synthetic lethality with *chk1* deletion, we checked the *swi7H4* and *chk1* delete *swi7H4* double mutant strains for their ability to form colonies at permissive temperature after transiently exposing them at non permissive temperature (36°C). The colony forming ability of *chk1* delete *swi7H4* double mutant was 4–5 time less as compared to *swi7H4* single mutant after 6hr exposure of the cells at 36°C (Fig 1B and 1C). The inability of the *swi7H4* mutant to form colonies in the *chk1* deletion background indicates that in the absence of normal checkpoint response the *swi7H4* mutant cells lose viability at a faster rate as compared to the cells having proficient checkpoint response.

### The double mutant of *swi7H4* with *chk1* deletion aggravates the chromosome segregation defects

**Fig 2:**
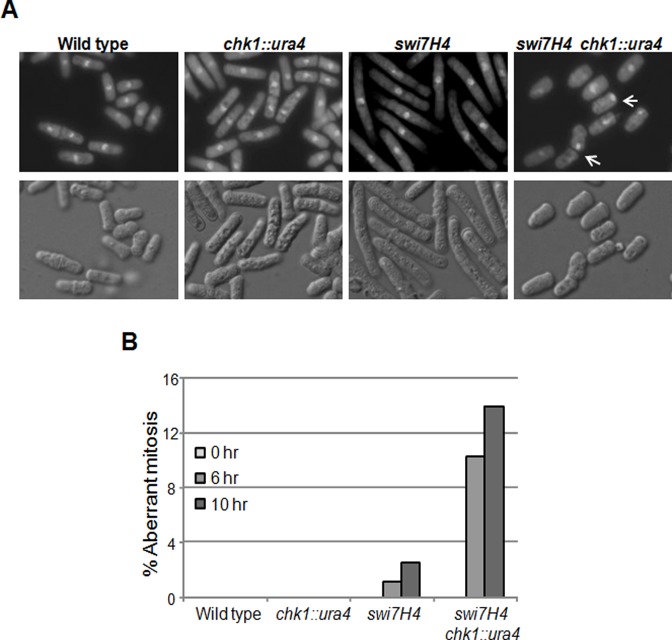
*swi7H4* mutant cells exhibit chromosome segregation defect at non permissive temperature. (A) Cells were grown at 25°C till mid log phase and then shifted at 36°C for 6 hr, fixed with 70% ethanol and stained with DAPI to visualize nuclei (upper panel) or with differential interference contrast (DIC) filter (lower panel). Arrow indicates defective chromosome segregation. (B) Indicated strains were grown till mid log phase at 25°C and then shifted at 36°C. Cells were collected at indicated time interval, fixed with 70% ethanol and stained with DAPI. Cells having aberrant chromosome segregation were counted.

The synthetic lethality of *swi7H4* mutant with *chk1* null mutant prompted us to look for the mitotic defects in the double mutant. Wild type, *chk1* delete, *swi7H4* and *chk1* delete *swi7H4* double mutant strains were grown at permissive temperature then shifted at 36°C. Samples were collected at 6hr and 10hr time points and cells were stained with DAPI. While wild type and *chk1* delete cells did not exhibit any chromosome segregation defects, an approximately 10 fold increase in the frequency of aberrant mitosis in *chk1* delete *swi7H4* double mutant (10%) as compare to *swi7H4* single mutant (1%) was observed after 6 hr at restrictive temperature (Fig 2A and 2B). Further incubation at non permissive temperature for 10hr results in about 14% aberrant mitosis in *chk1* delete *swi7H4* double mutant as compared to *swi7H4* single mutant cells that exhibit only 2.5% aberrant mitosis (Fig 2B). These results strengthen our observation that in the absence of a Chk1-dependent checkpoint response, the *swi7H4* mutant cells exhibit severe chromosome segregation defects.

### *Swi7H4* mutant cells delay the mitotic progression at non permissive temperature in a Chk1 dependent manner

**Fig 3:**
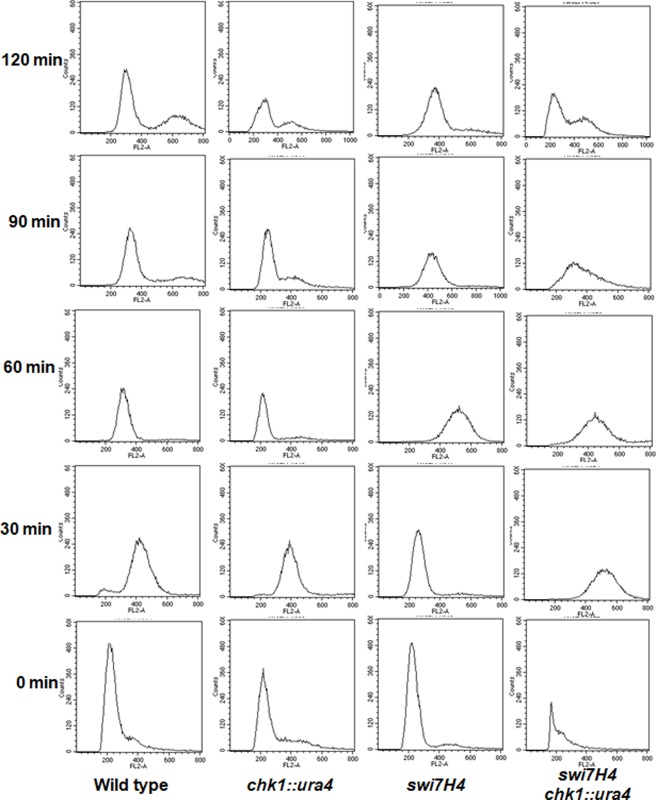
Mitotic progression was delayed in *swi7H4* mutant cells. Strains were grown till mid log phase, synchronized in early S phase by treating with 12mM hydroxyurea (HU) for 4 hr at 25°C, washed and released at 36°C. Samples were collected at 30 minute time interval fixed, stained with propidium iodide and FACS analysis was performed using BD FACS calibre for DNA content analysis.

In order to investigate the role of *swi7H4* mutant in checkpoint function we performed FACS analysis after releasing the cells from HU arrest at non permissive temperature. As shown in Fig 3 the peak of 2C DNA content was observed after 30 minutes in wild type and *chk1* delete cells while in *swi7H4* mutant cells the peak corresponding to 2C DNA content appear only after 60 minutes of release suggesting a mitotic delay in *swi7H4* mutant cells. Interestingly this mitotic delay was absent in *chk1* delete *swi7H4* double mutant as the peak of 2C DNA content appear 30 minutes after the release from HU arrest (Fig 3) indicating the requirement of Chk1 for the mitotic delay in *swi7H4* mutant.

**Fig 4:**
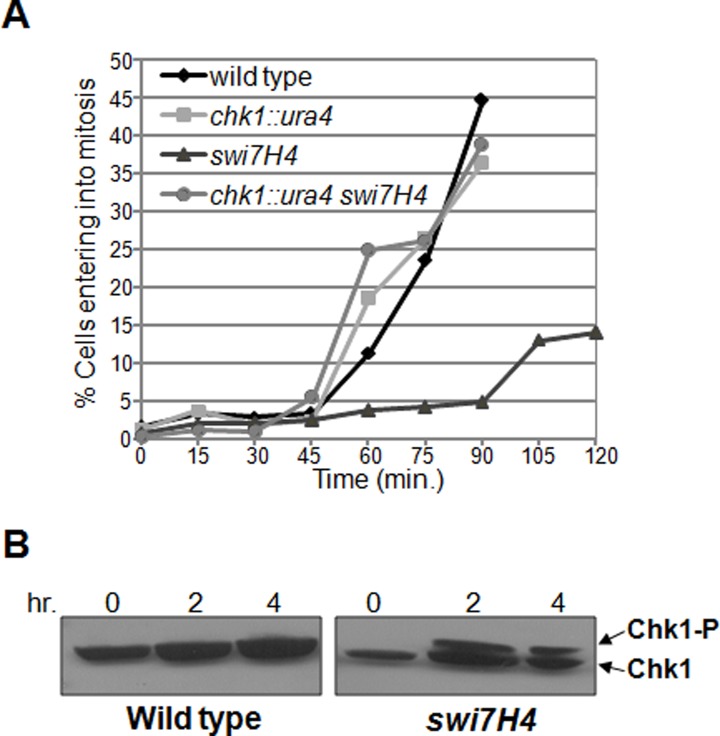
*swi7H4* mutant cells delay the mitotic entry and activates checkpoint kinase Chk1. (A) Cells were processed as in Fig 3, samples were collected at 15 minute interval and the number of cells having two nuclei was counted using DAPI as an indication of cells entering into mitosis. (B) Indicated strains were grown at permissive temperature till mid log phase then shifted at 36°C for indicated time. Protein lysate was prepared as described in Material and Methods, samples were run on 8% SDS PAGE, transferred on nitrocellulose membrane and probed with anti HA antibody.

To further investigate the mitotic delay in *swi7H4* mutant cells we monitored the appearance of binucleated cells as an indicator of mitotic progression as described earlier [27]. The cells were arrested in early S phase by growing them in 12mM HU for 4 hr then released at non permissive temperature and stained with DAPI to examine the nuclei. The *chk1* deletion and wild type cells enter mitosis at 45 minutes at non permissive temperature (Fig 4A) suggesting normal mitotic progression. In contrast, the *swi7H4* mutant cells begin to enter mitosis only at 90 minutes, with 13% of the cells bi-nucleating after 105 minutes shift at non permissive temperature (Fig 4A) suggesting a mitotic delay in *swi7H4* mutant cells. More importantly in *chk1* deletion background *swi7H4* mutant cells fail to delay mitotic entry (Fig 4A), suggesting that the *swi7H4* mutant cells generate a Chk1 dependent checkpoint response at non permissive temperature.

### *Swi7H4* mutant cells activates the checkpoint kinase protein Chk1

Activation of Chk1 in response to DNA damage results a decrease in mobility of Chk1 on SDS-PAGE [28]. Aggravated chromosomal segregation defect in the *swi7H4 chk1* delete mutant and delay in the mitotic progression in *swi7H4* mutant cells prompted us to question whether Chk1 protein get activated at non permissive temperature in *swi7H4* mutant background. The HA tagged Chk1 was introduced in *swi7H4* background by genetic crosses. Activation of Chk1 was monitored by western blotting using anti HA antibody (F7). We observed a slow migrating band of Chk1-HA in *swi7H4* mutant just after 2 hr incubation at non permissive temperature (Fig 4B, right panel). In contrast the wild type cells grown under same conditions do not exhibit this phenomenon (Fig 4B, left panel). These observations suggest that defects generated due to *swi7H4* mutation leads to the activation of checkpoint kinase protein Chk1 at non permissive temperature.

### The *swi7H4* mutant cells accumulate DNA damage at non permissive temperature

**Fig 5:**
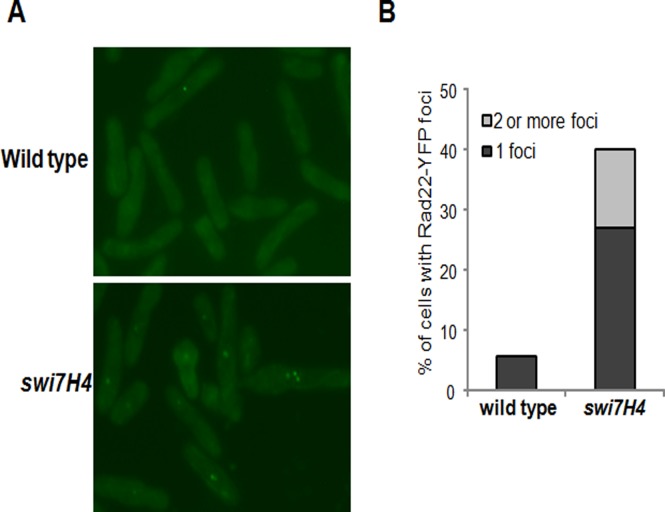
*swi7H4* mutant cells have elevated level of Rad22 YFP foci at non permissive temperature. (A) Indicated strains were grown till mid log phase at 25°C, shifted at non permissive temperature (36°C) for 6hr. The cells were processed for indirect immunofluorescence microscopy as described in *Materials and Methods*. (B) About 200 cells for each sample were counted and the percentage of cells containing Rad22-YFP foci was plotted.

Rad22, a homologue of budding yeast Rad52 [29] is required for homology-dependent double strand break repair and meiosis. During homologues recombination Rad22 binds to single stranded DNA that leads to the formation of Rad22-YFP foci [30]. We analyzed the appearance of Rad22 foci in *swi7H4* mutant at non permissive temperature. In comparison to wild type cells, *swi7H4* mutant cells exhibited eight fold increases in the level of Rad22-YFP foci at non permissive temperature (Fig 5A and 5B) suggesting that *swi7H4* mutant cells accumulate DNA damage at non permissive temperature even in the absence of exogenous DNA damaging agent.

### *Swi7H4* mutation exhibit synthetic lethality with genes involve in DNA repair pathway

**Fig 6:**
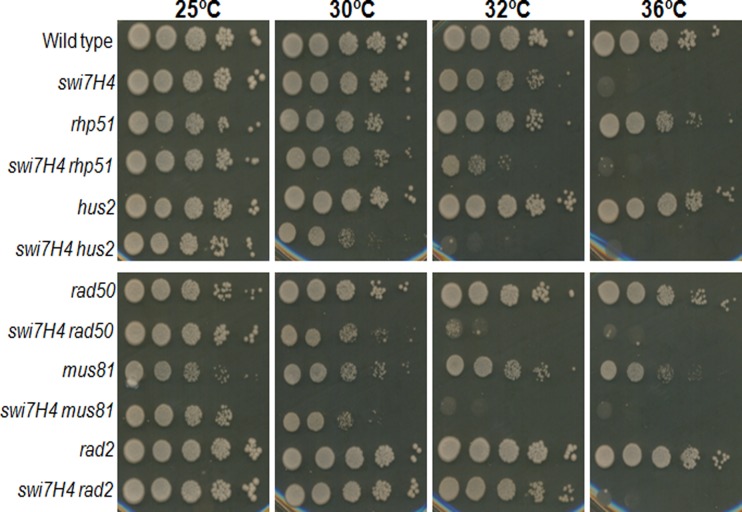
Genetic interaction studies of *swi7H4* mutant with genes involve in DNA repair pathway. Indicated strains were grown at 25°C, serially diluted and spotted on YEA plate. Plates were incubated at indicated temperature for 3–4 days before taking photographs.

Appearance of Rad22-YFP foci in *swi7H4* mutant strain suggests accumulation of DNA damage at non permissive temperature. These results prompted us to study the genetic interaction between *swi7H4* mutant allele and genes involved in DNA repair pathway. The double mutants of *swi7H4* with *rhp51*, *rad50*, *rqh1/hus2* and *mus81* knockout were constructed using standard genetic crosses and their survival was compared at semi and non permissive temperature. As presented in Fig 6A, the colony forming ability of double mutant of *swi7H4* with *rhp51*, *rad50*, *rqh1* and *hus2* delete cells was reduced as compared to the single mutants at semi permissive temperature (32°C) indicating the synthetic lethality between the *swi7H4* and genes involved in DNA repair pathway. In contrast, the *rad2* delete *swi7H4* double mutant cells were growing normally under the same condition (Fig 6A) suggesting that the absence of *rad2*, a Fen1 endonuclease which is required for the removal of RNA primer during DNA replication does not affect the survival of *swi7H4* mutant cells.

Cell cycle checkpoints are complex signal transduction pathways that involve sensors, transducers and effectors to block the cell cycle progression. In response to DNA damage the sensor kinase Rad3 and the effector kinase Chk1 play important role for the efficient and timely repair of the genome. DNA polymerase α is a well known component of DNA replication machinery required for lagging strand synthesis. Other than involvement in the initiation of DNA replication it is also found to be associated with the heterochromatin maintenance [13]. Here we tried to widen the characteristic behaviour of the *swi7H4*, a mutant allele of DNA polymerase α in DNA damage checkpoint response. The defects in *swi7H4* mutant results in DNA damage that delay mitosis with about eight fold increase in the Rad22 foci (Fig 4A). To further investigate the mechanism of mitotic delay in *swi7H4* mutant, we analyzed the activation of checkpoint kinase, Chk1. The 10 fold increase aberrant mitosis in the double mutant of *swi7H4* with *chk1* deletion and the activation of Chk1 at non permissive temperature suggests the activation of Chk1 dependent checkpoint response in *swi7H4* mutant cells. DNA polymerase α has been shown to play a critical role in coordinating S-phase with mitosis. Temperature sensitive DNA polymerase α mutant has been shown to perturb DNA replication and induces downstream kinase response [14,15]. At semi permissive temperature the *swi7H4* mutant allele of DNA polymerase α has been shown to exhibit higher mutation rate as compared to wild type [31,32]. We propose that the replication perturbation by *swi7H4* mutation could generate DNA damage that leads to the activation of checkpoint response. Subsequently in absence of DNA damage checkpoint protein (Chk1) or the protein responsible for DNA repair pathway the mutant cells exhibit severe defects that leads to synthetic lethal interaction.
